# Plasma Epstein-Barr virus DNA and risk of nasopharyngeal carcinoma in a prospective seropositive population

**DOI:** 10.1186/s12885-021-08408-0

**Published:** 2021-06-01

**Authors:** Wen-Jie Chen, Wen-Na Xu, Hai-Yun Wang, Xiao-Xia Chen, Xue-Qi Li, Shang-Hang Xie, Dong-Feng Lin, Su-Mei Cao

**Affiliations:** 1 Department of Cancer Prevention, Cancer Prevention Center, State Key Laboratory of Oncology in Southern China, Sun Yat-sen University Cancer Center, Guangzhou, China; 2 Department of Medicine Laboratory, Sun Yat-Sen University Cancer Center, Guangzhou, China; 3 Department of Pathology, Guangzhou Women and Children’s Medical Center, Guangzhou Medical University, Guangzhou, China; 4 Guangzhou Institute of Pediatrics, Guangdong Provincial Key Laboratory of Research in Structural Birth Defect Disease, Guangzhou Women and Children’s Medical Center, Guangzhou Medical University, Guangzhou, China; 5 School of Public Health, Sun Yat-Sen University, Guangzhou, China; 6 State Key Laboratory of Oncology in South China, Collaborative Innovation Center for Cancer Medicine, and Guangdong Key Laboratory of Nasopharyngeal Carcinoma Diagnosis and Therapy, Sun Yat-Sen University Cancer Center, Guangzhou, China

**Keywords:** Nasopharyngeal carcinoma, Plasma EBV DNA, Risk prediction, Population screening

## Abstract

**Objective:**

Plasma Epstein-Barr virus (EBV) DNA is considered a biomarker for nasopharyngeal carcinoma (NPC). However, its long-term role in NPC development is unclear.

**Materials and methods:**

A total of 1363 participants seropositive for EBV VCA-IgA and EBNA1-IgA in a community-based NPC screening program in southern China were tested for plasma EBV DNA levels by real-time qPCR between 2008 and 2015. New NPC cases were confirmed by active follow-up approach and linkage to local cancer registry through the end of 2016. Cox proportional hazards regression analysis was performed to calculate the hazard ratios (HRs) for NPC risk with plasma EBV DNA.

**Results:**

Thirty patients were newly diagnosed during a median 7.5 years follow-up. NPC incidence increased with the plasma EBV DNA load ranging from 281.46 to 10,074.47 per 100,000 person-years in participants with undetectable and ≥ 1000 copies/ml levels; the corresponding cumulative incidence rates were 1.73 and 50%. Furthermore, plasma EBV DNA loads conferred an independent risk for NPC development after adjustment for other risk factors, with HRs of 7.63 for > 3–999 copies/ml and 39.79 for ≥1000 copies/ml. However, the HRs decreased gradually after excluding NPC cases detected in the first 2 to 3 years and became statistically nonsignificant by excluding cases detected during the first 4 years.

**Conclusion:**

Elevated plasma EBV DNA can predict NPC risk over 3 years. Monitoring plasma EBV DNA can be used as a complementary approach to EBV serological antibody-based screening for NPC.

**Supplementary Information:**

The online version contains supplementary material available at 10.1186/s12885-021-08408-0.

## Introduction

Although nasopharyngeal carcinoma (NPC) is rare in most parts of the world, it is fairly frequent in Southeast Asia [1]. In essence, Epstein-Barr virus (EBV) infection is a necessary cause for approximately 95% of all NPC [2]. Primary EBV infection usually occurs in childhood and the virus preferentially infects within memory B cells in latency in healthy individuals [3]. Rarely, the virus is activated to an active lytic phase by endogenous and environmental stress. This transformation is regarded as the key step for NPC initiation and development, characterized by elevated viral DNA load and antibodies against multiple EBV antigens in circulation [4, 5]. According to the natural history studies for EBV, primary EBV infection occurs earlier in life in developing countries and is typically subclinical. EBV preferentially infects within memory B cells in latency in healthy individuals. The virus can be occasionally reactivated by endogenous and environmental stress, with increased aberrant virus reactivation and antibody responses against multiple EBV antigens, such as VCA-IgA, and EBNA1-IgA. Several long-term prospective studies have reported that elevated levels of antibodies against EBV antigens in serum precede several years of NPC development [6]. The proliferated B lymphocytes could release EBV participles and efficiently mediate cell-to-cell contact for EBV infection into the nasopharyngeal epithelial cells. Under constant attack from the virus, genetic instability increases and subsequently induces tumorigenesis in the susceptible individual. And then, latent EBV infection is established in the malignant nasopharyngeal epithelial cells [7].

Several prospective studies have convinced that elevated EBV-specific immunoglobulin A (IgA) antibodies are correlated with 4–21 fold risks for NPC and precede tumor detection by several years [8, 9]. Testing for serological EBV antibodies has therefore been established as the basis of NPC screening test in the endemic regions [10]. With the conveniences of cheap and easy to measure, they are usually be used as the primary screening markers to stratify NPC risk in NPC screening [11–13]. Among many EBV antibody testing, IgA antibodies against viral capsid antigen (VCA-IgA) and nuclear antigen 1 (EBNA1-IgA) have shown high sensitivity (> 90%) [14]. A community-randomized screening trial has been initiated in southern China to evaluate the efficacy of these two antibodies testing for early detection of NPC [11, 15, 16]. The current data have demonstrated a favorable effect with increased early detection rate and reduced NPC mortality in the screening participants [16]. However, the relatively low specificity (< 90%) of EBV serological antibody resulted in a reduced predictive value (PPV, ~ 5%), which lead to unnecessary psychological/financial burden for individuals without NPC. Therefore, the identification of new prediction factor as a complementary approach in EBV serological positive individuals is of interest.

Growing evidence in initial cross-sectional studies have revealed that plasma EBV DNA is a promising NPC indicator. The virus DNA load is also correlated with tumor burden, remission, and recurrence. However, a long-term relationship between plasma EBV DNA load and NPC risk is still lacking. In this study, we examined EBV DNA load in plasma from 1363 EBV-seropositive individuals in a community-based NPC screening program and prospectively evaluate its prediction value for NPC development over 5 years.

## Materials and methods

### Study population in the parent cohort

This study was based on a large community-based NPC screening program conducted in Sihui County, Guangdong province, southern China from 2008 [15]. The enrolled criteria include (1) residents aged 30–69 years; (2) without a history of NPC; and (3) with an Eastern Cooperative Oncology Group (ECOG) score of 0–2. For each participant, 6 mL of blood was obtained for testing the two screening markers of VCA-IgA (EUROIMMUN AG, Lübeck, Germany) and EBNA1-IgA (Zhongshan Bio-Tech Company, Zhongshan, China) by ELISA. The NPC risk scores were calculated by a risk prediction algorithm (Logit*P* = − 3.934 + 2.203*VCA-IgA + 4.797*EBNA-IgA) [11, 15], and three risk groups were stratified based on the predefined *P* scores (low-risk: < 0.65; medium-risk: ≥0.65- < 0.98; and high-risk: ≥0.98). Seropositive individuals were defined as those falling within either the serological high-risk or medium-risk groups.

At recruitment, all participants were asked to complete a structured questionnaire through face-to-face interviews by the trained researchers. The contents in the questionnaire included demographic information, such as sex, age, cigarette smoking, salted food intake, family history of NPC, and education level. Smokers were defined as having smoked at least one cigarette every 1–3 days during a 6-month period. The frequency of consumption of salted food at the time of the study interview was categorized into 2 groups: monthly or less, and weekly and more.

At the end of 2015, a total of 10,209 residents were tested for the screening markers and completed the questionnaire survey at baseline [17] . Written informed consent was obtained from each participant, and this study was approved by the Ethics Review Committee of the Sun Yat-sen University Cancer Center (NCT00941538, Clinical Trials.gov). This study was performed in accordance with the Declaration of Helsinki.

### Study population in the current cohort

**Fig. 1:**
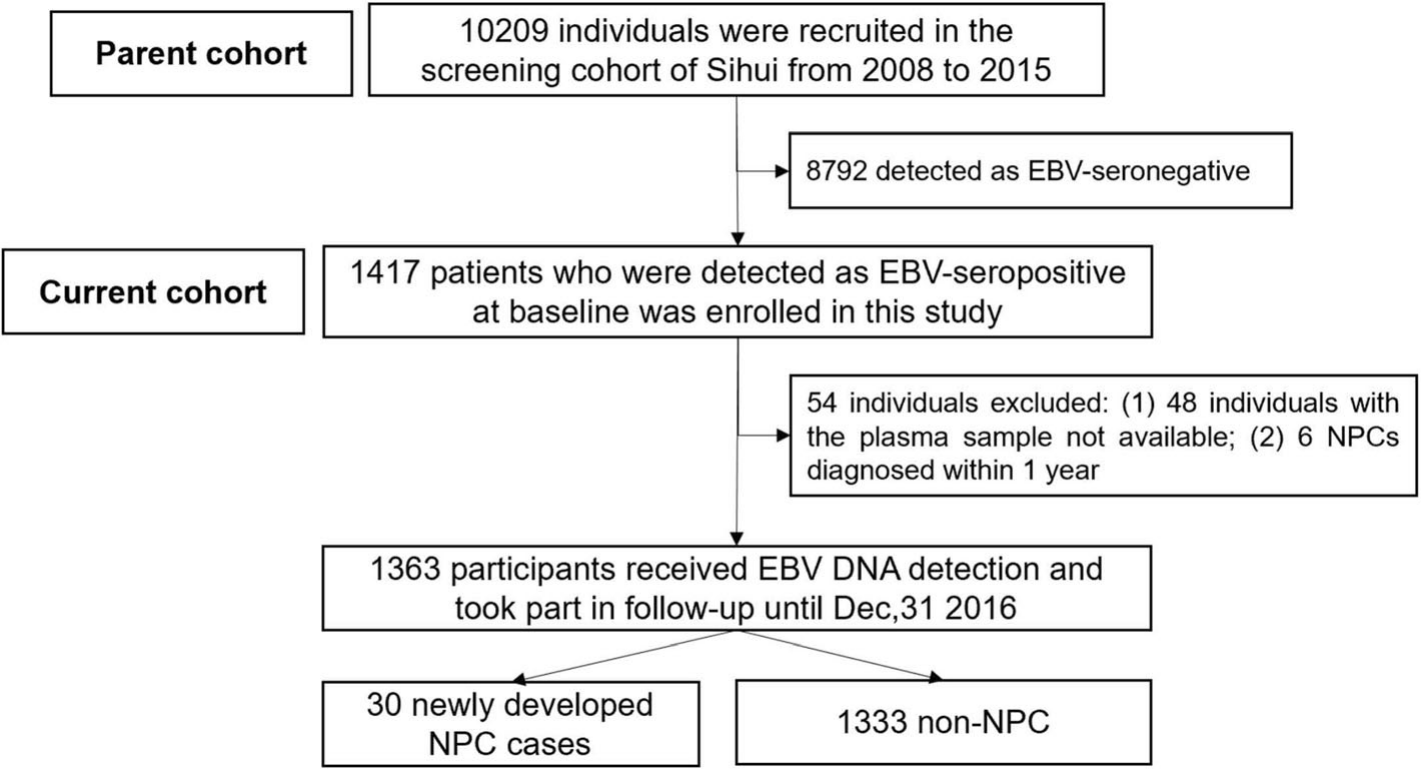
Flow chart of selecting screening participants

To evaluate the association between plasma EBV DNA load and NPC occurrence in EBV seropositive population, all seropositive participants with EBV antibody scores ≥0.65 (*n* = 1417) in the parent cohort were selected to test for plasma EBV DNA by real-time quantitative polymerase chain reaction (Fig. 1). Among them, 54 ineligible individuals were excluded, including 48 individuals not available for serum samples, 6 diagnosed with NPC within one year. Therefore, 1363 EBV seropositive subjects were recruited in the analysis, with 397 in the high-risk group and 966 in the medium-risk group.

### Real-time quantitative polymerase chain reaction (qRT-PCR)

Plasma DNA was extracted using the QIAamp Blood Kit (Qiagen, Hilden, Germany), and EBV DNA was detected within the BamHI-W region of the EBV genome by qRT-PCR [18]. The sequences of the primers used were as follows: 5′-GCCAG AGGTA AGTGG ACTTT-3′ (forward) and 5′-TACCA CCTCC TCTTC TTGCT-3′ (reverse). The dual fluorescently labeled oligomer probe sequence was 5′-(FAM) CACAC CCAGG CACAC ACTAC ACAT (TAMRA)-3′. This detection was performed with an Applied Biosystems 7500 Real-Time PCR System (Foster City, CA, USA). Cycle threshold (CT) values were defined as the number of cycles required for exceeding background level for the fluorescent signal. Samples were defined as undetectable if the CT values excess 38 cycles (the lowest limit of reliable quantification [LLOQ]), equal to 0–3 copies/ml of EBV DNA in plasma. Duplicate samples and multiple negative blanks were analyzed, and the mean quantity of each duplicate was used for further concentration calculations. The standard concentration ladders were set at 10^6^, 10^5^, 10^4^, 10^3^, and 10^2^ copies/ ml . The following equation was used to calculate the concentration of plasma EBV DNA: $\mathrm{C}=\mathrm{Q}\times \frac{V_{DNA}}{V_{PCR}}\times \frac{1}{V_{EXT}}$ (C: the target concentration in plasma, copies/ml; Q: the target quantity (copies) determined by a sequence detector in a PCR; V_DNA_: the total volume of DNA obtained after extraction; V_PCR_: the volume of DNA solution used for PCR; and V_EXT_: the volume of plasma/serum extracted).

### Ascertainment of incidence NPC and follow-up

Those in the serological high-risk group were referred to endoscopy examination for clinical evaluation. If suspicious lesions were observed in the endoscopy, nasopharyngeal biopsies were also performed. Pathologically diagnosed patients were immediately given advice for treatment. Those seropositive individuals also entered an accelerated follow-up group with annual screening. Those in the serological low-risk group were recommended for another screening with a 4–5 years interval. All participants were followed annually by linkages with local Cancer, Death and Population Registries. NPC cases were classified according to the World Health Organization (WHO) pathological classification system [19]. Among the 397 participants with EBV high-risk individuals, 247 received the endoscopy examinations and 50 further undertaken nasopharyngeal biopsies. At the end of 2016, 36 NPC cases were identified, with 6 in the first 1 year and 30 diagnosed after 1 year.

### Statistical analysis

The person-years of follow-up for each participant were calculated from the date of recruitment to the date of NPC diagnosis, death, emigration, or loss to follow-up or to December 31, 2016, whichever came first. After excluding the NPC cases that occurred in the first year, the annualized NPC incidence rate was calculated by dividing the number of incident NPC cases by the person-years of follow-up. The hazard ratios (HRs) and 95% confidence intervals (CIs) for NPC incidence among the different plasma EBV DNA level groups were calculated by Cox regression analysis adjusted for sex, age, education level, tobacco smoking, family history of NPC, and salted food intake. The cumulative incidences of NPC by plasma EBV DNA level were calculated by Kaplan-Meier plots and compared by the log-rank test. To control the potential bias in our evaluation of NPC risk by EBV DNA load, we analyzed three sub-cohorts by excluding NPC cases detected within the first 2, 3 and 4 years of follow-up. A two-sided test with a *P* value < 0.05 was considered statistically significant. All analyses were performed by using SAS 9.4 (SAS Institute Inc., Chicago, IL, USA) and R Language 3.5 software (http://www.rproject.org).

## Results

### Baseline characteristics

A total of 1363 EBV-seropositive participants were recruited to this study, and their baseline characteristics are presented in Table 1. Among them, 649 participants were males (47.62%), with a median age of 49 years (interquartile range [IQR], 43–57); 564 (41.38%) participants had a smoking history (ever and current smokers), and 192 participants (14.09%) had eaten salted food monthly or more. Moreover, 61 participants (4.48%) had a family history of NPC. A total of 875 participants (64.2%) had an education level of more than 6 years.

**Table 1:** Baseline characteristics of enrolled participants

Variables		n(%)
**Sex, n (%)**	male	649 (47.62%)
	female	714 (52.38%)
**Age, n (%)**	**Median age, years (IQR)**	49 (43 ~ 57)
	30–39	216 (15.85%)
	40–49	472 (34.63%)
	50–59	461 (33.82%)
	60–69	214 (15.70%)
**Smoking, n (%)**^a^	never	799 (58.62%)
	current or former	564 (41.38%)
**Salted food, n (%)**	monthly or less	1171 (85.91%)
	weekly and more	192 (14.09%)
**Family history of NPC, n (%)**	no	1302 (95.52%)
	yes	61 (4.48%)
**Education, n (%)**	≤6 year	488 (35.8%)
	> 6 year	875 (64.2%)
**EBV-based risk score, n (%)**^b^	0.65 - < 0.98	966 (70.87%)
	≥0.98	397 (29.13%)

^a^Smoking was defined as ever having smoked at least one cigarette every 1–3 days during a 6-month period; ^b^EBV-based risk score were defined by a predefined logistic regression model

### NPC incidence and cumulative incidence by plasma EBV DNA load

Based on the tested plasma EBV DNA levels, only 2.86% (39/1363) of seropositive individuals were detectable for plasma EBV DNA. Through December 31, 2016, with a median of 7.5 years (IQR, 4.2–8.2 years) of follow-up, 30 NPC cases were identified after excluding 6 NPC cases detected in the first year (Table 2). NPC incidence rate in EBV DNA detectable participants (2878.70 per 100,000 person-years) was approximately 10-fold higher than that in EBV DNA undetectable participants (281.46 per 100,000 person-years), (*P* < 0.05). Furthermore, the incidence rates per 100,000 person-years increased gradually from 2239.01 in the individuals with plasma EBV DNA load > 3–999 copies/ml to 10,074.47 in those with the load ≥1000 copies/ml (*P* < 0.001).

**Table 2:** Univariate and multivariable Cox analyses for risk factors of NPC

Variables	Participants(n)	Person-Years	NPC Cases(n)	Incidence Rate Per 100,000 Person-Years	Crude HR(95%CIs)	***P-value***	Fully Adjusted HR(95%CIs)^b^	***P-value***
**Sex**								
male	649	4009.16	18	448.97	Reference		Reference	
female	714	4405.70	12	272.37	0.611 (0.294–1.269)	0.186	1.381 (0.413–4.617)	0.601
**Age**								
30–49	688	4470.52	15	335.53	Reference		Reference	
50–69	675	3944.34	15	380.29	1.099 (0.537–2.250)	0.796	0.832 (0.389–1.783)	0.637
**Smoking**								
never	799	4929.13	11	223.16	Reference		Reference	
current or former	564	3485.73	19	545.08	2.415 (1.149–5.076)	0.020	3.566 (1.055–12.060)	0.041
**Salted food**								
monthly or less	1171	7073.05	25	353.45	Reference		Reference	
weekly and more	192	1341.81	5	372.63	1.050 (0.402–2.745)	0.921	0.867 (0.329–2.288)	0.774
**Family history**								
no	1302	8025.25	25	311.52	Reference		Reference	
yes	61	389.61	5	1283.34	4.227 (1.618–11.043)	0.003	2.838 (1.029–7.826)	0.044
**Education**								
≤ 6 year	488	2967.21	14	471.82	Reference		Reference	
> 6 year	875	5447.65	16	293.70	0.638 (0.312–1.308)	0.220	0.433 (0.191–0.982)	0.045
**EBV DNA (copies/ml)**								
≤ 3(undetectable)	1324	8171.70	23	281.46	Reference		Reference	
> 3 (detectable)	39	243.16	7	2878.70	10.476 (4.495–24.414)	< 0.0001	10.049 (4.227–23.887)	< 0.0001
> 3–999	35	223.31	5	2239.01	8.123 (3.088–21.370)		7.627 (2.83–20.558)	
≥ 1000	4	19.85	2	10,074.47	37.763 (8.890–160.414)	< 0.0001^a^	39.789 (8.406–188.348)	< 0.0001

^a^P trend for the comparision among EBV DNA undetectable group, DNA levels at > 3–999 copies/ml and DNA levels ≥1000 copies/ml

^b^ Maximum adjustment: all the aforementioned variables were used for adjustment including sex, age, smoking status, NPC family history, salted food,education level, EBV DNA

**Fig. 2:**
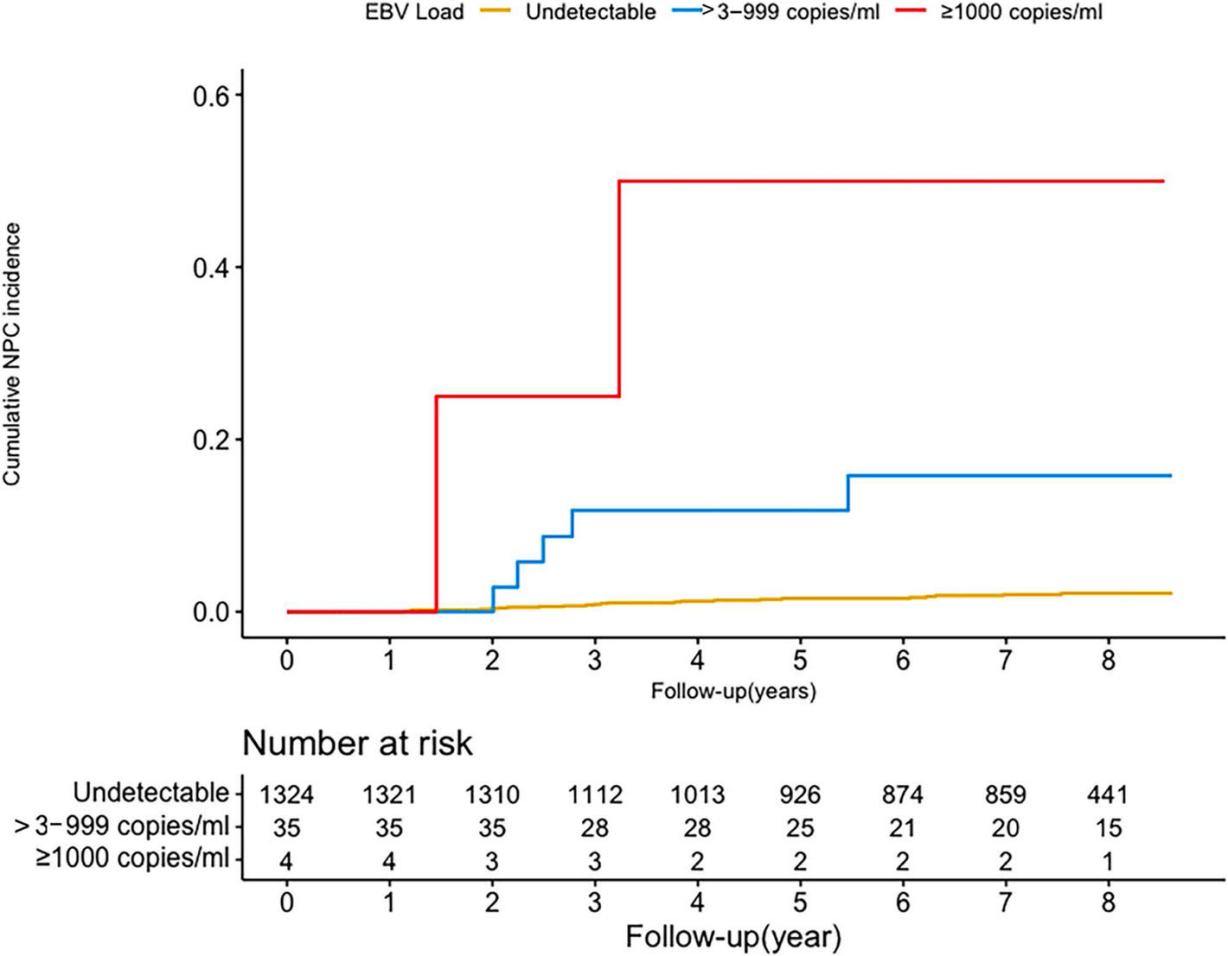
Cumulative incidence of nasopharyngeal carcinoma by plasma EBV DNA levels. The cumulative incidence of NPC in the 1363 participants. The log-rank test shows that higher levels of plasma EBV DNA present higher risks of NPC development. **a** EBV DNA positive vs. undetectable (*P* < 0.001). **b** Comparison between EBV DNA undetectable, > 3 ~ 999 copies/ml, and ≥ 1000 copies/ml (*P* < 0.001)

Figure 2 shows the cumulative incidences of NPC by plasma EBV DNA level during the follow-up period. There was a biological gradient trend of the cumulative NPC incidence with plasma EBV DNA levels increasing (*P* < 0.05). Approximately 50% of individuals with plasma EBV DNA loads ≥1000 copies/ml developed NPC during the follow-up period, compared with 14.29% of those with > 3–999 copies/ml and 1.74% with undetectable plasma EBV DNA loads (both *P < 0*.001). When we chose 500 copies/ml and 1500 copies/ml as the highest cutoff values, the same trends were found in the three grade groups with EBV DNA levels (both *P* < 0.001) (Supplementary Table 1).

### NPC risk by plasma EBV DNA load

The NPC risks associated with different plasma EBV DNA levels are shown in Table 2. Compared with participants with undetectable EBV DNA levels, those with EBV DNA loads of > 3–999 copies/ml and ≥ 1000 copies/ml had crude HRs of 8.123 (95% CI: 3.088–21.370; *P* < 0.0001) and 37.763 (95% CI: 8.890–160.414; *P* < 0.0001), respectively. After full adjustment for potential risk factors, including age, sex, smoking, family history of NPC, education, and salted food intake, the same trend was found, with *P* < 0.0001. Meanwhile, we found that smoking, family history of NPC, and education level were also significantly associated with NPC development, and the adjusted HRs were of similar magnitude, as seen in multiple regression analyses (Table 2).

### NPC risk by plasma EBV DNA level considering different follow-up durations

To our knowledge, plasma EBV DNA in NPC patients may originate from tumor cells. To minimize the potential reverse causality, we further evaluated the association of NPC risk with plasma EBV DNA levels after excluding all new cases diagnosed in the first 2, 3 or 4 years. Compared to the HR of NPC risk in participants with undetectable EBV DNA, a gradual decrease in HRs with detectable plasma EBV DNA was observed after excluding cases diagnosed in the first 2 (10.878, 95% CI: 4.215–28.072; *P* < 0.0001) or 3 years (5.139, 95% CI: 1.136–23.252; *P* = 0.0336). Moreover, the HR of NPC risk was further decreased to 4.054 (95% CI: 0.500–32.845; *P* = 0.1898) after excluding cases diagnosed in the first 4 years and became statistically nonsignificant (Table 3).

**Table 3:** Predictive value of EBV DNA for NPC development by different follow-up duration

Variables	> 2 years		> 3 years		> 4 years	
	Adjusted HR(95%CIs)	***P-value***	Adjusted HR(95%CIs)	***P-value***	Adjusted HR(95%CIs)	***P-value***
≤3(undetectable)	Reference		Reference		Reference	
> 3 (detectable)	10.878 (4.215–28.072)	< 0.0001^a^	5.139 (1.136–23.252)	0.0336^a^	4.054 (0.500–32.845)	0.1898^a^
> 3–999	9.637 (3.491–26.607)	< 0.0001^b^	2.785 (0.359–21.619)	0.3273^b^	4.472 (0.552–36.257)	0.3739^b^
≥1000	30.111 (3.662–247.587)		33.560 (3.996–281.872)		–	

^a^HRs are adjusted by sex, age, smoking status, NPC family history, salted food,education level; ^b^ P trend for the comparision among EBV DNA undetectable group, DNA levels at > 3–999 copies/ml and DNA levels ≥1000 copies/ml

## Discussion

As plasma EBV DNA has been shown to be a promising indicator of NPC tumor presence and is widely used to predict NPC prognosis in clinical practice [15], we further evaluate its risk prediction value in the EBV seropositive individuals in an NPC screening cohort. Our prospective cohort study provides compelling evidence that plasma EBV DNA load is strongly associated with an increased risk of NPC over 3 years. The association is independent of several potential confounders, including age, sex, education level, family history of cancer, cigarette smoking, salted food intake. Moreover, the higher virus DNA load revealed a gradual increasing risk for NPC, with the cumulative NPC incidence increased gradually from 1.74% in individuals with plasma EBV DNA undetectable to 14.29% in those with > 3–999 copies/ml and 50% with ≥1000 copies/ml.

To the best of our knowledge, this study is the largest and longest prospective follow-up study on the association between plasma EBV DNA loads and NPC outcomes. All the evidence in this study suggest plasma EBV DNA can be used as a complementary approach to EBV serological antibody-based screening for NPC. Closer clinical monitoring and even more sensitive diagnostic examination should be recommended for these extremely high-risk individuals with both circulating EBV DNA and antibodies positive, especially in the first several years. Since magnetic resonance imaging (MRI) examination of nasopharynx has shown higher sensitivity to capture minor, early-stage NPC difficult to visualize by endoscopy and to maximize NPC detection within EBV-based NPC screening programs, using plasma EBV DNA as an auxiliary approach can minimize false seropositive screening test results and reduce cost burden associated with unnecessary referrals [20].

However, plasma EBV-DNA testing is not ideal as a primary screening tool. Even we utilized a cutoff at the LLOQ, only 21.8% NPC cases with EBV seropositive are detectable by plasma EBV DNA in this cohort, that is nearly 80% NPC cases would be missed. Similar to our result, several previous population-based cross-sectional screening studies have also reported the sensitivity of plasma EBV DNA (56.4–73.0%) is inferior to EBV antibody testing alone (> 90%) in NPC screening [21].

Our study has strengths and limitations. This study was based on a large population-based prospective screening cohort with a long-term follow-up and designed specifically to evaluate EBV-based serological screening for the early detection of NPC. In addition, the EBV seropositive population received active follow-up and comprehensive clinical examinations, thus largely reducing diagnostic bias and errors in this cohort. Nevertheless, several limitations to this study should also be mentioned. First, although this study was based on a longitudinal cohort, we did not retest and monitor the fluctuation pattern for EBV DNA during the follow-up period. We don’t know how many percentages of the participants had transient or chronic EBV infection at the time of testing. Monitoring the fluctuation pattern of plasma EBV DNA and NPC occurrence in a large cohort with longer follow-up is still needed in the future. Moreover, the association between plasma EBV DNA load and NPC onset was only investigated in a high-risk area, hence, our results might not be applicable to other areas. More prospective studies are warranted to confirm our findings in other regions.

In summary, we evaluate plasma EBV DNA loads and the risk of NPC development in a large-scale NPC screening program in an endemic area. This study indicates that plasma EBV DNA can predict an increased risk of NPC over 3 years and be used as a complementary approach to EBV serological antibody-based screening to improve NPC screening efficiency.

## Supplementary Information

**Additional file 1.**

## Data Availability

Data and materials of this work are available from the corresponding author on reasonable request.

## Abbreviations

NPC: Nasopharyngeal Carcinoma
EBV: Epstein-Barr Virus
IgA: Immunoglobulin A
HR: Hazard Ratios
CIs: Confidence Intervals
IQR: Interquartile Range
